# Taxonomy of two synnematal fungal species from *Rhus chinensis*, with *Flavignomonia* gen. nov. described

**DOI:** 10.3897/mycokeys.60.46395

**Published:** 2019-10-31

**Authors:** Ning Jiang, Qin Yang, Ying-Mei Liang, Cheng-Ming Tian

**Affiliations:** 1 The Key Laboratory for Silviculture and Conservation of the Ministry of Education, Beijing Forestry University, Beijing 100083, China; 2 Forestry Biotechnology Hunan Key Laboratories, Central South University of Forestry and Technology, Changsha 410004, China; 3 Museum of Beijing Forestry University, Beijing Forestry University, Beijing 100083, China

**Keywords:** Diaporthales, Gnomoniaceae, systematics, taxonomy

## Abstract

*Rhus chinensis* represents a commercially and ecologically important tree species in China, but suffers from canker diseases in Jiangxi Province. Synnemata, pycnidia and ascomata were discovered on cankered tissues. Strains were obtained from single ascospore or conidium within the fruiting bodies and identified based on morphological comparison and the phylogenetic analyses of partial ITS, LSU, *tef1* and *rpb2* gene sequences. As a result, two species were confirmed to represent two kinds of synnemata. One of these species is described herein as *Flavignomonia rhoigena***gen. et sp. nov.**; and *Synnemasporella aculeans* is illustrated showing ascomata, pycnidia and synnemata. *Flavignomonia* is distinguished from *Synnemasporella* by the colour of the synnematal tips. Additionally, *Flavignomonia* can be distinguished from the other gnomoniaceous genera by the formation of synnemata.

## Introduction

Many Diaporthales species are important branch canker pathogens, forming acervuli or pycnidia on diseased tissues (Rossman et al. 2007, Senanayake et al. 2017, Jiang et al. 2018, 2019, Wijayawardene et al. 2018, Yang et al. 2018, Voglmayr et al. 2019). However, two diaporthalean species with synnemata were reported to cause cankers, namely *Synnemasporella aculeans* (syn. *Cryptodiaporthe aculeans*) and *S. toxicodendri* (Fan et al. 2018). These two species differ from the other diaporthalean taxa in conidiomata and form a distinct clade phylogenetically, which was named Synnemasporellaceae and distinguished by Fan et al. (2018).

Gnomoniaceae was initially introduced with *Gnomonia* as the type (Winter 1886). Species in Gnomoniaceae formed upright perithecia, with or without long or short necks and presence or absence of stromatic tissues (Barr 1978, Sogonov et al. 2008, Walker et al. 2012). In the recent monograph of Diaporthales, 34 genera were accepted in the family Gnomoniaceae (Senanayake et al. 2018). Subsequently, *Neognomoniopsis* and *Tenuignomonia* were added based on both molecular and morphological evidence (Crous et al. 2019, Minoshima et al. 2019).

Chinese gall (*Rhus chinensis* Mill.) has a range of uses as source of medicine, dye and oil, and has a wide distribution in China (Wang et al. 2014). However, cankers were found to be associated with different ascomata during our fungal collection trips in Jiangxi Province, China. The objectives of the present study were to identify these fungi based on morphological and phylogenetic evidence.

## Materials and methods

### Sample collections and isolation

We conducted our fungal collection surveys from April to October in China, and found *Rhus chinensis* to be one of the major tree species in Jiangxi Province. Twigs, branches and stems were carefully checked, and diseased tissues were cut into small pieces and packed in paper bags. Isolates were obtained by transferring the ascospores or conidial masses from ascomata to sterile PDA plates, incubating at 25 °C until spores germinated. Single germinating spores were transferred onto new PDA plates, which were kept at 25 °C in darkness. Specimens were deposited in the Museum of the Beijing Forestry University (**BJFC**) and axenic cultures maintained in the China Forestry Culture Collection Centre (**CFCC**).

### Morphological analysis

Recognition and identification of the fungal species on *Rhus chinensis* was based on fruiting bodies formed on the bark. Ascomata and conidiomata were sectioned by hand using a double-edged blade, and microscopic structures were observed under a dissecting microscope. At least 10 conidiomata/ascomata, 10 asci, and 50 conidia/ascospores were measured to calculate mean and standard deviation. Measurements are reported as maxima and minima in parentheses and the range representing the mean plus and minus the standard deviation of the number of measurements given in parentheses (Voglmayr et al. 2017). Microscopy photographs were captured with a Nikon Eclipse 80i compound microscope equipped with a Nikon digital sight DS-Ri2 high definition colour camera, using differential interference contrast illumination. Nomenclatural novelties and descriptions were deposited in MycoBank (Crous et al. 2004).

### DNA extraction, PCR amplification and sequencing

Genomic DNA was extracted from colonies grown on cellophane-covered PDA plates using a modified CTAB method (Doyle and Doyle 1990). PCR amplifications were performed in a DNA Engine Peltier Thermal Cycler (PTC-200; Bio-Rad Laboratories, Hercules, CA, USA). The primer sets ITS1/ITS4 (White et al. 1990) were used to amplify the ITS region. The primer pair LR0R/LR5 (Vilgalys and Hester 1990) was used to amplify the LSU region. The primer pairs EF1-688F/EF1-986R or EF1-728F/TEF1-LLErev (Carbone and Kohn 1999; Jaklitsch et al. 2005; Alves et al. 2008) were used to amplify *tef1* gene. The primer pair dRPB2-5f/dRPB2-7r (Voglmayr et al. 2016) was used to amplify the *rpb2* gene. The polymerase chain reaction (PCR) assay was conducted as described by Fan et al. (2018). PCR amplification products were assayed via electrophoresis in 2 % agarose gels. DNA sequencing was performed using an ABI PRISM 3730XL DNA Analyzer with a BigDye Terminater Kit v.3.1 (Invitrogen, USA) at the Shanghai Invitrogen Biological Technology Company Limited (Beijing, China).

### Phylogenetic analyses

The preliminary identities of the isolates sequenced in this study were obtained by conducting a standard nucleotide BLAST search using the sequences generated from the above primers of the different genomic regions (ITS, LSU, *tef1* and *rpb2*). The BLAST results showed that three isolates were grouped in the family Gnomoniaceae, and five isolates in the genus *Synnemasporella*. The phylogenetic analyses for the three gnomoniaceous isolates were conducted based on Senanayake et al. (2018), supplemented by sequences of *Tenuignomonia styracis* and *Neognomoniopsis quercina* from Crous et al. (2019) and Minoshima et al. (2019). *Melanconis marginalis* (CBS 109744) in Melanconidaceae was selected as the out-group taxon. All sequences were aligned using MAFFT v. 6 (Katoh and Toh 2010) and edited manually using MEGA v. 6 (Tamura et al. 2013). Phylogenetic analyses were performed using PAUP v. 4.0b10 for maximum parsimony (MP) analysis (Swofford 2003), and PhyML v. 3.0 for Maximum Likelihood (ML) analysis (Guindon et al. 2010).

MP analysis was run using a heuristic search option of 1000 search replicates with random additions of sequences with a tree bisection and reconnection algorithm. Other calculated parsimony scores were tree length (TL), consistency index (CI), retention index (RI), and rescaled consistency (RC). ML analysis was performed using a GTR site substitution model including a gamma-distributed rate heterogeneity and a proportion of invariant sites (Guindon et al. 2010). The branch support was evaluated using a bootstrapping method of 1000 replicates (Hillis and Bull 1993). The matrix was partitioned for the different gene regions. Phylograms were shown using FigTree v. 1.4.3 (Rambaut 2016). Novel sequences generated in the current study were deposited in GenBank (Table 1) and the aligned matrices used for phylogenetic analyses in TreeBASE (accession number: S25047).

**Table 1.** Strains used in the phylogenetic tree and their culture accession and GenBank numbers. Strains from this study are in bold.

**Species**	**Strains**	**GenBank numbers**			
		**ITS**	**LSU**	***tef1***	***rpb2***
*Alnecium auctum*	CBS 124263	KF570154	KF570154	KF570200	KF570170
*Ambarignomonia petiolorum*	CBS 116866	EU199193	AY818963	NA	EU199151
	CBS 121227	EU254748	EU255070	EU221898	EU219307
*Amphiporthe tiliae*	CBS 119289	EU199178	EU199122	NA	EU199137
*Anisogramma anomala*	529478	EU683064	EU683066	NA	NA
*Anisogramma virgultorum*	529479	EU683062	EU683065	NA	NA
*Apiognomonia errabunda*	AR 2813	DQ313525	NG027592	DQ313565	DQ862014
*Apiognomonia veneta*	MFLUCC 16-1193	MF190114	MF190056	NA	NA
*Apioplagiostoma populi*	858501	KP637024	NA	NA	NA
*Asteroma alneum*	CBS 109840	EU167609	EU167609	NA	NA
*Asteroma* sp.	Masuya 8Ah9-1	NA	AB669035	NA	NA
*Cryptosporella hypodermia*	CBS 116866	EU199181	AF408346	NA	EU199140
*Discula destructiva*	MD 254	AF429741	AF429721	AF429732	NA
*Ditopella biseptata*	MFLU 15-2661	MF190147	MF190091	NA	MF377616
*Ditopella ditopa*	CBS 109748	DQ323526	EU199126	NA	EU199145
*Ditopellopsis* sp.	CBS 121471	EU254763	EU255088	EU221936	EU219254
***Flavignomonia rhoigena***	**CFCC 53118**	**MK432674**	**MK429917**	NA	**MK578102**
	**CFCC 53119**	**MK432675**	**MK429918**	NA	**MK578103**
	**CFCC 53120**	**MK432676**	**MK429919**	NA	**MK578104**
*Gnomonia gnomon*	CBS 199.53	DQ491518	AF408361	EU221885	EU219295
	CBS 829.79	AY818957	AY818964	EU221905	NA
*Gnomoniopsis alderdunensis*	CBS 125680	GU320825	NA	NA	NA
*Gnomoniopsis chamaemori*	CBS 803.79	EU254808	EU255107	NA	NA
*Gnomoniopsis racemula*	AR 3892	EU254841	EU255122	EU221889	EU219241
*Mamianiella coryli*	BPI 877578	EU254862	NA	NA	NA
*Marsupiomyces quercina*	MFLUCC 13-0664	MF190116	MF190061	NA	NA
*Marsupiomyces epidermoidea*	MFLU 15-2921	NA	MF190058	NA	NA
*Melanconis marginalis*	CBS 109744	EU199197	AF408373	EU221991	EU219301
*Neognomoniopsis quercina*	CBS 145575	MK876399	MK876440	NA	NA
*Occultocarpon ailaoshanense*	LCM 524.01	JF779849	JF779853	NA	JF779856
	LCM 522.01	JF779848	JF779852	JF779862	JF779857
*Ophiognomonia melanostyla*	LCM 389.01	JF779850	JF779854	NA	JF779858
*Ophiognomonia vasiljevae*	AR 4298	EU254977	EU255162	EU221999	EU219331
*Plagiostoma aesculi*	AR 3640	EU254994	EU255164	NA	EU219269
*Linospora capreae*	CBS 372.69	NA	AF277143	NA	NA
*Pleuroceras oregonense*	AR 4333	EU255060	EU255196	EU221931	EU219313
*Pleuroceras pleurostylum*	CBS 906.79	EU255061	EU255197	EU221962	EU219311
*Phragmoporthe conformis*	AR 3632	NA	AF408377	NA	NA
*Valsalnicola oxystoma*	AR 5137	JX519561	NA	NA	NA
	AR 4833	JX519559	JX519563	NA	NA
*Sirococcus tsugae*	AR 4010	EF512478	EU255207	EU221928	EU219289
	CBS 119626	EU199203	EU199136	EF512534	EU199159
*Synnemasporella aculeans*	CFCC 52094	MG682086	MG682026	MG682066	MG682046
***Synnemasporella aculeans***	**CFCC 53123**	**MK432679**	**MK429920**	**MK578148**	**MK578105**
	**CFCC 53124**	**MK432680**	**MK429921**	**MK578149**	**MK578106**
	**CFCC 53125**	**MK432681**	**MK429922**	**MK578150**	**MK578107**
	**CFCC 53126**	**MK432682**	**MK429923**	**MK578151**	**MK578108**
	**CFCC 53127**	**MK432683**	**MK429924**	**MK578152**	**MK578109**
*Synnemasporella toxicodendri*	CFCC 52097	MG682089	MG682029	MG682069	MG682049
*Tenuignomonia styracis*	BPI 89278	NA	LC379288	LC379282	LC379294

## Results

### Phylogenetic analyses

**Figure 1.**
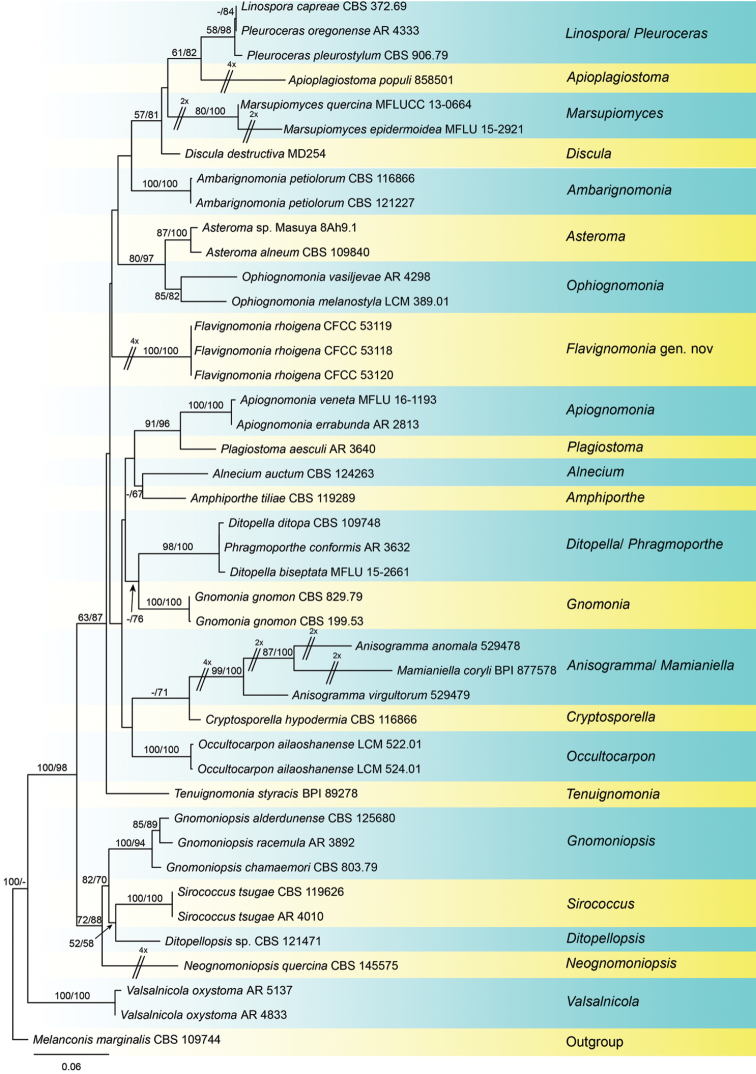
Phylogenetic tree based on an ML analysis of a combined DNA dataset of ITS, LSU, *tef1* and *rpb2* gene sequences for all genera with DNA data and some species of Gnomoniaceae. Bootstrap values ≥ 50 % for MP and ML analyses are presented at the branches. The scale bar represents the number of changes per site.

The alignment based on the combined sequence dataset (ITS, LSU, *tef1*, and *rpb2*) included 42 in-group taxa and one out-group taxon, comprising 3368 characters in the aligned matrix. Of these, 2201 characters were constant, 224 variable characters were parsimony-uninformative and 943 characters were parsimony informative (282 from the ITS-LSU, 280 from *tef1*, 381 from *rpb2*). The MP analysis resulted in nine equally most parsimonious trees with identical tree backbone. The best ML tree (lnL = −20604.0384) was compatible with the MP strict consensus tree, except for unsupported clades in Fig. 1. As the trees obtained from different analytical methods were similar, only the ML tree was present in Fig. 1. The phylogram based on the four gene sequence matrix indicated that the three strains from the present study represent a novel genus in Gnomoniaceae.

### Taxonomy

#### Flavignomonia

Taxon classificationFungiDiaporthalesSynnemasporellaceae

C.M. Tian, Q. Yang & N. Jiang gen. nov.

DAD63D30-357D-53BE-8FEA-5EBACB4FB8AA

829530

##### Diagnosis.

*Flavignomonia* is distinguished from *Synnemasporella* by the orange tips of its synnemata.

##### Type species.

*Flavignomonia rhoigena* C.M. Tian & Q. Yang

##### Etymology.

The generic name is derived from the colour of synnemata (flavus = yellow) and the genus name *Gnomonia*.

##### Description.

Sexual morph: not observed. Asexual morph: Conidiomata synnematal. Synnemata long and determinate, growing from host tissue, with brown base and orange tip, straight to curved, parallel, with flat to slightly concave and dark zone of conidiogenous cells and host tissue at their bases. Conidiophores reduced to conidiogenous cells. Conidiogenous cells phialidic, aggregated, hyaline, straight to curved, cylindrical, arranged adjacent to one another at the end of the synnema, producing a single conidium. Conidia cylindrical to oblong, smooth, multiguttulate, hyaline.

##### Notes.

*Flavignomonia* is included in Gnomoniaceae based on DNA sequences data. *Flavignomonia* is morphologically similar to *Synnemasporella* in forming synnemata (Wehmeyer 1933, Fan et al. 2018). However, *Flavignomonia*, typified with *Flavignomonia rhoigena*, is distinguished from *Synnemasporella* species by its orange synnematal tips and hyaline conidia (Fan et al. 2018).

#### Flavignomonia rhoigena

Taxon classificationFungiDiaporthalesSynnemasporellaceae

C.M. Tian & Q. Yang sp. nov.

7F7D0EAD-2C04-5A91-B125-2DF50F71C843

829531

Figure 2

##### Diagnosis.

*Flavignomonia rhoigena* can be distinguished from other gnomoniaceous species by the formation of synnemata.

##### Etymology.

Named after the host genus, *Rhus*.

##### Description.

Sexual morph: not observed. Asexual morph: Conidiomata synnematal. Synnemata (650–)750–1100 µm high, 150–300 µm diam, determinate, growing from host tissue, with brown base and orange tip, straight to curved, parallel, with flat to slightly concave and dark zone of conidiogenous cells and host tissue at their bases. Conidiophores reduced to conidiogenous cells. Conidiogenous cells (12.5–)16–22(–25) × 2 μm, phialidic, aggregated, hyaline, straight to curved, cylindrical, arranged adjacent to one another at the end of the synnema, producing a single conidium. Conidia cylindrical to oblong, smooth, multiguttulate, hyaline, (5–)5.5–7(–8) × 1.5–2 µm.

##### Culture characters.

On PDA at 25 °C in darkness, initially white, becoming olive-green to black after 3 wk, zonate with 3–4 well defined zones. Conidiomata distributed concentrically over agar surface.

##### Specimen examined.

CHINA, Jiangxi Province, Ganzhou City, Xunwu County, 24°52'31.34"N, 115°35'39.53"E, on branches of *Rhus chinensis*, 14 May 2018, Q. Yang, Y. Liu & Y.M. Liang (holotype BJFC-S1766, ex-type living cultures CFCC 53118, CFCC 53119 and CFCC 53120).

##### Notes.

*Flavignomonia rhoigena* is the type species of *Flavignomonia* in the family Gnomoniaceae. It can be easily distinguished from the other gnomoniaceous genera by its unique conidiomata (Walker et al. 2004, Senanayake et al. 2018, Crous et al. 2019, Minoshima et al. 2019).

**Figure 2.**
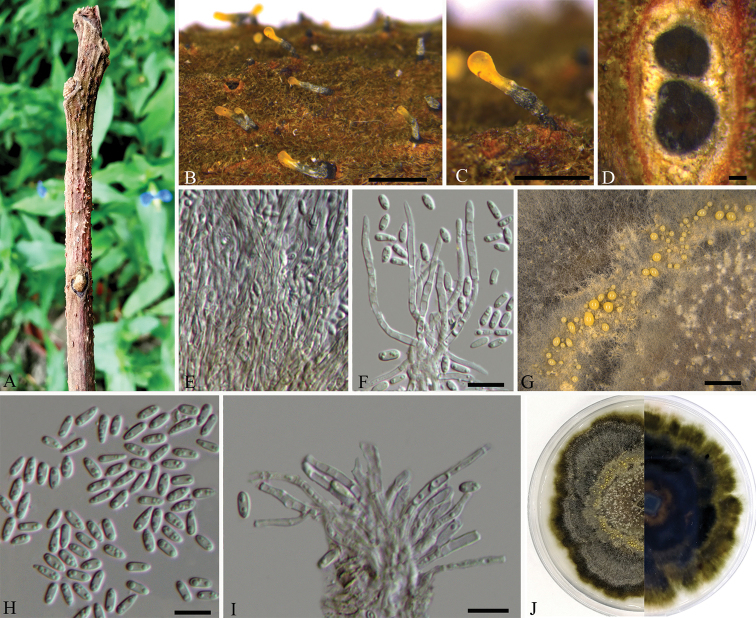
*Flavignomonia rhoigena* on *Rhus chinensis* (BJFC-S1766, holotype) **A–C** habit of conidiomata on twigs **D** transverse section through synnema **E** longitudinal section through synnema **F, I** conidiogenous cells attached with conidia **G** conidiomata on PDA **H** condia **J** the colony on PDA. Scale bars: 1 mm (**B**); 500 μm (**C**); 100 μm (**D**); 10 μm (**F, H–I**); 200 μm (**G**).

#### Synnemasporella aculeans

Taxon classificationFungiDiaporthalesSynnemasporellaceae

(Schwein.) X.L. Fan & J.D.P. Bezerra, Persoonia 40: 130. 2018.

C8503EB3-6F7E-529D-BB7A-97E1F130DB8E

Figure 3 , 4

##### Description.

Sexual morph: See Wehmeyer (1933) and Fan et al. (2018). Asexual morph: See Fan et al. (2018).

##### Specimens examined.

CHINA, Jiangxi Province, Ganzhou City, Xunwu County, 24°52'31.34"N, 115°35'39.53"E, on branches of *Rhus chinensis*, 14 May 2018, Q. Yang, Y. Liu & Y.M. Liang (BJFC-S1740, living culture CFCC 53123); Ganzhou City, Fengshan forest park, 25°44'32.14"N, 114°59'25.54"E, on branches of *Rhus chinensis*, 15 May 2018, Q. Yang, Y. Liu & Y.M. Liang (BJFC-S1753, living culture CFCC 53124 and CFCC 53125). 24°38'38.18"N, 115°33'58.45"E, on branches of *Rhus chinensis*, 16 May 2018, Q. Yang, Y. Liu & Y.M. Liang (BJFC-S1745, living culture CFCC 53126 and CFCC 53127).

##### Notes.

*Synnemasporella aculeans* was proposed as a new combination in the new genus *Synnemasporella* based on the description of *Cryptodiaporthe aculeans* (Fan et al. 2018), which was introduced producing perithecial ascomata, and an asexual morph producing sporodochial and/or pycnidial conidiomata (Wehmeyer 1933). In the present study, five isolates from canker tissues on *Rhus chinensis* were congruent with *S. aculeans* based on morphology and DNA sequences data. This was the first time that the sexual morph of *Synnemasporella aculeans* in China had been collected.

**Figure 3.**
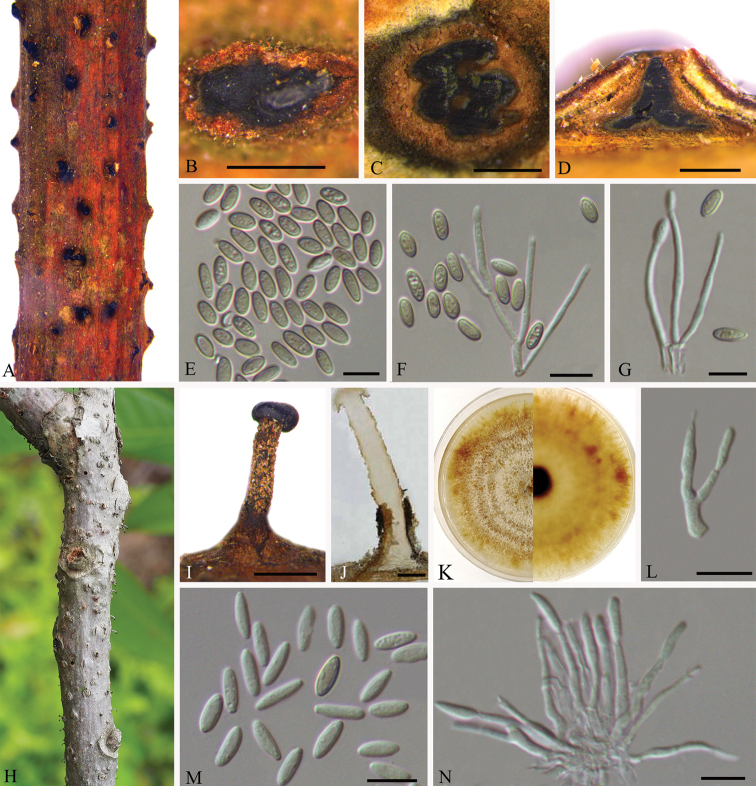
Asexual morphology of *Synnemasporella aculeans* on *Rhus chinensis* (BJFC-S1740) **A, B** habit of pycnidia on twigs **C** transverse section of pycnidium **D** longitudinal section through pycnidium **E** conidia **F, G** conidiogenous cells and conidia **H, I** habit of synnemata on twigs **J** longitudinal section through synnema **K** the colony on PDA **L, N** conidiogenous cells bearing conidia **M** conidia. Scale bars: 500 μm (**B–D, I, J**); 10 μm (**E–G, L–N**).

**Figure 4.**
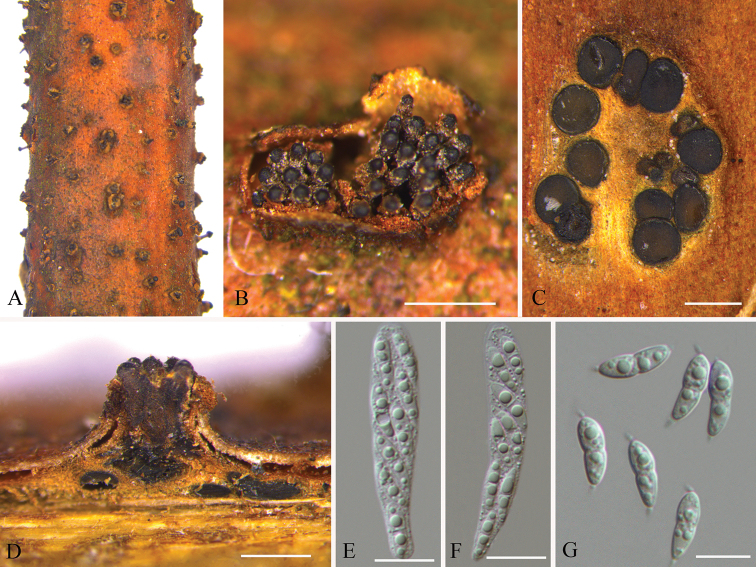
Sexual morphology of *Synnemasporella aculeans* on *Rhus chinensis* (BJFC-S1745) **A, B** habit of ascomata on twigs **C** transverse section of ascomata **D** longitudinal section through ascomata **E, F** asci **G** ascospores. Scale bars: 500 μm (**B–D**); 10 μm (**E–G**).

## Discussion

In this study, two diaporthalean species forming synnemata on *Rhus chinensis* were identified based on morphology and ITS, LSU, *tef1*, and *rpb2* sequence datasets. As a result, *Flavignomonia* typified with *F. rhoigena* is proposed as a new genus in Gnomoniaceae for its distinct phylogenic position and distinctive asexual fruiting body, Also, *Synnemasporella aculeans* strains were successfully isolated from perithecia, pycnidia and synnemata, which was confirmed by molecular data.

Nineteen fungal species have been recorded from the commercially and ecologically important tree species in China, including *Cladosporium cladosporioides*, *Cronartium quercuum*, *Mycosphaerella fushinoki*, *Pestalotiopsis diospyri*, *P. guepinii*, *P. mangiferae*, *P. sorbi*, *Phaeoramularia rhois*, *Phyllactinia corylea*, *Ph. rhoina*, *Pileolaria klugkistiana*, *Pi. shiraiana*, *Pseudocercospora rhoina*, *Ps. toxicodendri*, *Septoria* sp., *Tubercularia phyllophila*, *Uncinula verniciferae*, and two synnematal species from branch cankers in this study (Farr and Rossman 2019). *Flavignomonia rhoigena* and *Synnemasporella aculeans*, described and illustrated in the present study can be easily recognized by the asexual fruiting bodies, and they differ from each other in the colour of the synnematal tips.

Gnomoniaceae is a globally distributed fungal family on diverse plant hosts (Mejía et al. 2008, 2011a, 2011b, 2012, Sogonov et al. 2008, Walker et al. 2012, Senanayake et al. 2017, 2018). Host specificity of this family has been confirmed to be important in the evolution (Walker et al. 2014). Our newly discovered genus *Flavignomonia* was only found on *Rhus chinensis*, and more *Flavignomonia* species might be collected from the plant family Anacardiaceae in the future.

## Supplementary Material

XML Treatment for Flavignomonia

XML Treatment for Flavignomonia rhoigena

XML Treatment for Synnemasporella aculeans
